# Perceptions of Community-Dwelling Patients and Their Physicians on OxyContin® Discontinuation and the Impact on Chronic Pain Management

**DOI:** 10.1155/2017/5402915

**Published:** 2017-01-30

**Authors:** Feng Chang, Sara Ibrahim

**Affiliations:** School of Pharmacy, University of Waterloo, Waterloo, ON, Canada

## Abstract

OxyContin, formerly one of the most commonly prescribed medications for chronic pain in Canada, was discontinued, delisted from the Ontario Drug Formulary, and replaced by a tamper-resistant formulation in 2012. The impact of discontinuing OxyContin on patients formerly prescribed it to treat chronic pain was unreported. Patients with chronic pain aged 45 years and over (*n* = 13) were recruited from two primary care and one specialty practice sites and interviewed using a semistructured guide to capture their experiences with discontinuing OxyContin, the efficacy of alternate medications, and relationships with physicians. Additional interviews were conducted with their physicians (*n* = 7) to obtain physician perceptions on discontinuation and to expand understanding of the patients' experiences. Aspects of patients' pain and medical care through the discontinuation process revealed emergent themes that both converge and diverge from that of treating physicians. Areas of divergence include the motive for discontinuation, which was condemned by most patients but supported by all physicians, and the perceived impact of discontinuance on pain control, with the majority of patients experiencing a negative impact and most physicians describing it as insignificant. Perceptions of patients and physicians coincided on the need to optimize pain management practices.

## 1. Introduction

OxyContin, a controlled-release semisynthetic formulation of oxycodone, was approved by the US Food and Drug Administration in 1995 [1] to treat moderate to severe chronic pain when continuous analgesia was required [1–3]. This opioid analgesic entered the Canadian market in 1996 [4] and the Ontario Provincial Drug Benefit Formulary in 2000 [5, 6]. OxyContin was made as a controlled-release formulation, but the tablet could be crushed and then injected or snorted to produce a strong sense of euphoria [7–10]. It became one of the most highly abused medications in Canada [11] and the United States [7, 12, 13]. In Ontario, prescriptions for oxycodone medications increased by 900% from 1991 to 2009 [5, 14], and the number of oxycodone-related deaths increased by 416% between 1999 and 2004 [5]. Across the country, a 2010 report shows that Canada has been ranked the second biggest per capita consumer of prescription opioids, with 460,000 doses ingested daily per million people [15]. According to Industry Canada, sales of OxyContin rose from $1 million in 1998 to $217 million in 2010, indicating an annual increase of 9.1% [5, 16]. In the past two decades, overdose deaths involving opioids among Ontarians have increased by 2.5 times reaching about 50 deaths annually by 2010 [17]. The majority (79.7%) of these deaths were accidental or of undetermined cause.

OxyContin was discontinued and delisted from the Ontario Drug Benefit Formulary in early 2012 [18]. The decision was touted as a positive step towards curbing prescription opioid misuse. However, the change also coincided with the expiry of OxyContin's patent and OxyContin maker Purdue Pharma's decision to phase out OxyContin in favour of OxyNEO®, a tamper-resistant formulation of the same medication. OxyNEO was more difficult to crush or liquefy, making it harder to snort or inject. Since OxyNEO was deemed bioequivalent to OxyContin, it was approved for market without further studies required. OxyNEO was made available for patients previously on OxyContin for a one-year period (February 2012 to 2013), and since then, OxyNEO has remained available in Ontario when paid for through private drug plans, out of pocket, as a part of the Exceptional Access Program or through Palliative Care Facilitated Access [19]. In the same year, Health Canada also approved six generic versions of OxyContin [20].

Complicating the picture further, the risks of addiction associated with OxyContin and the ensuing discontinuation were frequently covered in the media while the drug's usefulness in chronic pain management remained debatable. A subsequent systematic review showed that, despite widespread use, there was insufficient evidence to suggest long-term opioids were actually effective in improving chronic pain and function [21]. Working through the waves of change and finding a balance between adequate treatment and negative outcomes were significant challenges for patients with chronic pain and their prescribers. However, very little has been written about their reflections as social agents in this discontinuation process. This study aims to explore the perceptions of patients with chronic pain and their physicians on OxyContin discontinuation and the impact that it had on chronic pain care and management.

## 2. Methods

A concurrent nested mixed-methods approach was used to capture the experiences of patients with chronic pain and their physicians after OxyContin discontinuation. This study was reviewed and approved by the Office of Research Ethics at the University of Waterloo and the Health Sciences Research Ethics Board at Western University.

### 2.1. Patient and Physician Recruitment

Patients were recruited from client bases of patients who had received OxyContin for chronic pain at two rural primary care sites and one urban specialty pain clinic in Southwestern Ontario. Recruitment posters and flyers included brief information on the research topic, study purpose, inclusion criteria, time requirements, and contact details. Eligibility criteria included being 45 years or older, suffering from chronic pain for at least six consecutive months, and having taken OxyContin for at least six months before discontinuation. Eligibility was determined by telephone screening. The age of 45 years was selected as a cut-off to target people middle-aged and older since the prevalence of chronic pain increases with age [22–24].

Names of respective physicians responsible for OxyContin prescribing were collected from study patients. The physicians were contacted by the research team, informed about the study, and invited to participate.

All participants were assigned random code identifiers to maintain confidentiality.

### 2.2. Data Collection

Data were collected between July and October 2013. Study interviews were held either in a private clinic room or at home for participating patients and at the clinic for participating physicians. Before interviews, patients completed two questionnaires—the Patient Treatment Satisfaction Scale (PTSS) and the Brief Pain Inventory (BPI)—to provide point-in-time insights into measures such as pain intensity, satisfaction with current pain medication, and effect of pain on their quality of life (QOL).

The PTSS, used to evaluate satisfaction with pain treatment, is comprised of 39 items assembled in five domains: (a) information about pain and its treatment (five items); (b) medical care (eight items); (c) impact of current pain medication (eight items); (d) side effects of current pain medication (12 items); and (e) satisfaction with current pain medication [divided into medication characteristics (three items) and efficacy (three items)] to be evaluated on a five-point Likert scale [25].

The BPI, applicable to pain of different etiologies [26, 27], includes four pain severity items, body diagrams to specify the location of pain, a question about percentage of pain relief attained by medication, and seven pain interference items. Patients rated their current pain intensity and strength in the last 24 hours at its worst, least, and average on 0–10 scales. Patients also rated the extent of pain interference with seven QOL domains: (a) general activity, (b) mood, (c) walking ability, (d) work, (e) relations with others, (f) sleep, and (g) enjoyment of life.

Patient interviews took about 40–60 minutes and physician interviews took about 20–30 minutes. Each followed a guide covering the discontinuation process, pain relief, and personal experiences. Physician participants were not informed of any information relevant to the patient participant and interviews focused on their general perceptions instead of specific cases. All interviews were conducted by an investigator who was a graduate student in pharmacy with training in qualitative research.

### 2.3. Data Analysis

Quantitative survey data were analyzed using Qualtrics, an online survey analysis application.

The audio recordings of each interview were transcribed manually and proofread by a second reader and then sent to participants for review. Data from the interviews and personal observations were sorted and manually coded for units of meaning using a two-step process, where an initial four-to-five-word code was assigned to each line of the transcript to reflect the meaning of the line. These codes were reviewed repeatedly to elucidate specific focused codes and to synthesize theoretical categories representing distinct patterns and themes. The data sets were compared and contrasted to identify similarities and differences. Memo-writing, both field notes made during the interview and reflexive memos written during the coding and categorizing of data, was employed to capture reflections on the collected data. The themes that emerged were then categorized into focused classes. Analysis and theme categorization were confirmed by a second reader with Ph.D. training in qualitative research.

## 3. Results

### 3.1. Participant Characteristics

A total of six patients enrolled from the urban specialty clinic and seven enrolled from the two rural primary care sites (four and three, resp.). Participant characteristics are summarized in Table 1. The mean age was 58 years (range: 46–78 years). On average, they had suffered from chronic pain for 7 years (range: 2–11 years).

All patients provided their physician's contact information, resulting in communications with eight physicians (five general practitioners (GPs) and three pain specialists) at three locations. All physicians enrolled in the study, but one GP withdrew, leaving one patient unrepresented.

### 3.2. Patient Treatment Satisfaction Scale

The PTSS revealed that eight (61.5%) patients received sufficient information on causes of their pain and treatment options, seven (53.8%) required more information on their pain medications, and ten (76.9%) wished to be more informed on possible side effects. None wanted to receive less information on any aspect. Twelve patients (92.3%) noted that they questioned their physicians easily, eleven (84.6%) agreed that medical staff kept them from worrying, and ten (76.9%) confirmed their physicians' willingness to prescribe desired medications. Patients were divided on their opinions of the impact of their medication on their QOL, with equal numbers agreeing or disagreeing that their medication improves concentration or permits increased participation in leisure activities. However, eight patients (61.5%) agreed that their medication positively affected their physical health and improved their outlook on life but nonetheless failed to meet expectations, while nine (69.2%) were dissatisfied with their current medications. Only three (23%) patients were satisfied with their medications, and one (8%) was neutral.

### 3.3. Brief Pain Inventory

Table 2 summarizes patient responses to the BPI questionnaire. Eleven patients (84.6%) reported their back as the most significant source of pain. Patients rated their current pain level at a mean value of 5.6 (range: 0–9, *n* = 13), with average pain at 5.2 (range: 1–8), which was relieved 53.8% (range: 10–90%) in the past 24 hours. Pain interfered most with sleep, normal work, and enjoyment of life, but other QOL domains were also negatively influenced by pain. Appetite was the only domain where some patients reported no interference.

### 3.4. Emergent Themes from Patient Interviews

The six major themes and their contributing subthemes that emerged are described below and listed in Table 3.

#### 3.4.1. Theme 1: Disagreement with the Motive for Discontinuation

Overall, patients strongly disagreed with the rationale for discontinuing OxyContin to reduce addiction. In contrast, patients strongly believed that discontinuation would fail to halt OxyContin addiction because people could instead abuse other medications, succeed in overcoming the tamper-resistant mechanism of the new formulation, and/or access OxyContin where it was still available. Patients felt they were being punished for the actions of people who misuse the medication and were concerned about being perceived as being dependent themselves. They also feared removing OxyContin would lead to increased emergency visits because of withdrawal, as well as increased pharmacy robberies and higher crime rates.

That kinda got me mad, cause I thought well you know…they're taking it off the market because of people abusing it…It's not fair to us, you know. … I think the government was wrong to… pull them off the market, you know, because of people abusing them, no like they weren't looking at the people that need them…But I think it's really unfair that people that really do need them can't get them. (Female, 53 years old, lower back pain for 6 years, average pain score was 6 out of 10)

Furthermore, patient participants felt neglected and inadequately considered in the decision to discontinue the drug. Some patients put blame on prescribers who prescribed opioids unprofessionally or too easily, thus contributing to the misuse problem. Others expressed uncertainty of the expertise of GPs in prescribing opioids such as OxyContin. Patients also condemned the approval and introduction of generic forms of OxyContin.

#### 3.4.2. Theme 2: Discontinuation Negatively Impacted Pain Control

While all thirteen patients reported successful pain management with OxyContin, eight were dissatisfied with OxyNEO, saying it provided reduced pain relief. OxyNEO was also felt to be slower in onset of action and more difficult to swallow.

I just found it just wasn't working for me…I went to the doctor and I talked to him and I said, look, I don't think this is working so…he just never filled out no more, no more prescriptions for me, cause if it's not working, why take it?…Um…I found the OxyContin worked so much faster where the other, I don't know, it just didn't, it just didn't cut the pain. I don't know why the difference was, if they were the same pill and that, but it just wouldn't, I didn't find like I was getting the relief out of it like I did the others. (Female, 54 years old, 10-year chronic back and leg pain, average pain score was 8 out of 10)

Other medications patients tried also failed to match the pain relief experienced with OxyContin and negatively impacted physical activity and cognitive ability. As a result, patients felt threatened by the discontinuation and deprived of their medication and resented enduring trials to optimize alternate medication regimes. Some experienced withdrawal while switching to other medications and preferred to continue with OxyContin, which they felt provided optimal benefit. Two patients resented having to pay for OxyNEO, which was not funded as a general benefit under the Ontario drug benefit program once the initial year ended.

I was very upset. That's what I was. People who worked and had insurance companies that would pay for it can continue it. We are the lower part of the income scale. My option is OxyNEO, but I have to pay for it. I have no idea how much it is and if I have to pay for my occupational therapy, if I have to pay for my, what's its name to come and do my exercises, if I have to pay for, I mean this country that pays for your medication is not telling the truth. (Female, 77 years old, chronic lower back pain for 8 years, average pain score was 5 out of 10)

#### 3.4.3. Theme 3: Discontinuation Insignificantly Impacted Pain Control

Five patients were unaffected by discontinuation, three received satisfactory pain control with their alternate medication, and two experienced similar residual pain while taking OxyContin.

It hasn't affected me at all. In other words, I wouldn't know, uh, whether I am, by the way I feel, I can't tell, I wouldn't be able to tell whether I am still taking OxyContin, if I was in a blind taste test, not taste test but uh, blind medication administration test I suppose. (Female, 78 years old, osteoarthritis pain for 4 years, average pain score was 1 out of 10)

Those with good pain control to begin with described a more smooth transition to OxyNEO (*n* = 2) or hydromorphone (*n* = 1). Both patients that experienced residual pain with OxyContin continued to have similar pain with OxyNEO.

#### 3.4.4. Theme 4: Choosing to Get Off OxyContin Permanently

Two patients decided to discontinue OxyContin by themselves because of concerns over becoming physically and psychologically dependent on it. Both suffered withdrawal symptoms and uncontrolled pain, diminishing their level of activity.

I realized that it was an addiction and I couldn't, I realized how I felt if I couldn't get to it…so I knew it was an addiction, and I knew I had to get off of them because that's not, my plan of life to live on addiction…so I decided that's it, I'm gonna do it on my own and I did. (Female, 68 years old, spinal stenosis for 11 years, average pain score was 5 out of 10)

Some other patients indicated their desire to discontinue opioids at some point in their lives, disliking the idea of lifelong reliance on a medication. Many expressed a fear of addiction but also feared withdrawal symptoms and intolerable pain from suspending use of opioids.

#### 3.4.5. Theme 5: Learning to Live with Pain

Seven patients' hopes for attaining optimally managed pain changed over time, replaced with an understanding that they will live with some degree of uncontrolled pain.

I just found different ways to cope with it, and to, I guess accept that, it's here to stay, and um, you just have to kind of figure out a way to, um, to try manage it and try, still have a bit of a life too…I guess the change just has to come from you just trying I mean myself just trying to, um, find a way through it or work around it. (Female, 52 years old, osteoarthritis and fibromyalgia pain for 10 years, average pain score was 7 out of 10)

Most participants felt the need to make additional pain medications available to chronic pain sufferers, as they had exhausted alternative medications and therapies. Participants voiced hopes for better alternatives and wished for the return of OxyContin.

#### 3.4.6. Theme 6: Barriers and Opportunities in Optimizing Care

All thirteen patients perceived flaws in patient education, communication, and treatment in the course of discontinuing OxyContin. They learned about OxyContin's discontinuation from the media and Internet. Some reported that their physicians did not provide information on why OxyContin was discontinued or the range of alternative medications available, revealing communication gaps partially attributable to difficulty in accessing physicians. When they visited their physicians, patients were disappointed by the reaction to their clearly communicated concerns.

The OxyNEOs…the doctor didn't even know side effects of it, the pharmacist told me side effects of it, the doctor didn't know. It's like she, here's a prescription, but I do not know nothing about it. (Female, 49 years old, diabetic neuropathy pain for 7 years, average pain score was 2 out of 10)

No, no, nobody said anything. Well, I asked my doctor and only because I heard on the news, she didn't come to me and tell me this was gonna happen, it was all the news…I wanted to know why, and um, I have to think, because I was very concerned, um, because at the time when I listened, when I heard it on the news, they were, they wanted to take it right off the market, that's what I was concerned about…I was worried what's the next step…like there was a lot of thoughts, uh, like that that was going through my mind. (Female, 63 years old, chronic pain caused by car accident for 8 years, average pain score was 6 out of 10)

Some patients recalled being put on OxyContin without receiving any information on the drug properties and its long-term effects in advance. One patient expressed a lack of trust in the medication-related information provided by her physician at discontinuation because the physician had failed to inform her about OxyContin's properties at initiation.

Patients also expressed uncertainty about the risk-benefit ratio of using OxyContin or alternative medications. Decisions were based upon their personal experiences and on their physicians' advice. Conflicting opinions among physicians and poor integration of medical services across healthcare professionals led to increased confusion about the medications. Less frequently mentioned concepts included learning about inaccurate information about medications, recalling bad experiences with healthcare providers, and self-medicating according to personal needs.

### 3.5. Emergent Themes from Physician Interviews

Seven physicians (four GPs and three pain specialists) provided insights on the discontinuation of OxyContin, which resulted in five themes, each with a number of subthemes. These are outlined in Table 4.

#### 3.5.1. Theme 1: Support for the Motive for Discontinuation

All physicians strongly supported the discontinuation of OxyContin and its removal from the Ontario Drug Benefits Formulary. Six supported the provincial government's decision to leave OxyNEO unfunded, thereby restricting accessibility through the Exceptional Access Program. They believed the addiction potential of OxyContin exceeded that of other opioids, resulting in elevated rates of misuse.

I think it was a good idea, to discontinue OxyContin, um, mainly because especially in the area where I practice we saw a lot of abuse and misuse, and still do, um, and given that there are other, you know, effective alternatives in terms of pain management, I think it was a good decision. (MD4)

#### 3.5.2. Theme 2: Discontinuation Insignificantly Impacted Pain Control

All physicians believed that discontinuation of OxyContin had insignificant influence on patients' ability to attain equivalent pain relief, as other pain medications were of equivalent efficacy. They asserted that transitions were carried out efficiently with a sufficient range of treatment options.

I don't really think I had any problems at all with my patients going from OxyContin to OxyNEO. (MD6)

It didn't seem like a big blip or anything in my practice, it just, patients were explained, they changed over and the pain was controlled, so it was not a big issue. (MD5)

Some seized the opportunity to switch patients to other medications because of their specific dislike of OxyContin. Two physicians perceived an association between unsatisfactory outcomes to illegitimate use of the medication by patients and expressed concerns for patients who found switching to a different painkiller problematic, stating that equivalent relief should be obtained with other medications, particularly OxyNEO.

One pain specialist who saw the highest number of patients in the clinic categorized 65%–75% of patients as ones who understood the reason behind the switch and were confident with switching to non-OxyNEO alternative medications. This further supported the perception that discontinuation had an insignificant impact on pain management.

#### 3.5.3. Theme 3: Sufficient Resources for Opioid Prescribing

Six physicians deemed existing guidelines for opioid use to be adequate and sufficient for physicians to prescribe opioids safely. They felt the use of opioids across different practice settings can be optimized when physicians follow the guidelines in place, ensure appropriate prescribing, and perform accurate conversions between opioids.

I think the Canadian guidelines for safe and effective opioid prescribing, if applied uniformly, will do a lot. (MD3)

Lack of adherence to the practice guidelines was thought to be the issue in prescribing. While a need to regulate opioid prescribing was not suggested, one GP mentioned the idea of a narcotic database, expecting that such an initiative would enhance monitoring strategies for opioid prescribing across the province.

#### 3.5.4. Theme 4: Disapproval of Generics

Physicians considered existing pain medications sufficient to replace OxyContin. All but one strongly opposed the introduction of generic products, believing that doing so would defeat the purpose of discontinuing OxyContin. No physician participant had prescribed generics to their patients and six actively discouraged their use. While they agreed generic products could be beneficial for some patients, this consideration was felt to be greatly outweighed by the negative consequences that could arise.

I'm actually very angry, what was the whole point then? What was the purpose of OxyNEO then? Right? That makes me mad…so the whole exercise about taking it off the street is actually negated if they have a generic on the market, right? (MD5)

I have never used a generic controlled release and I would not recommend it…it's a mimic of, uh, of OxyContin, and carries the same risks of substance abuse. So most pain specialists were against, the, release of generic controlled release oxycodone, and we discouraged the use, uh, and I don't use it. (MD2)

#### 3.5.5. Theme 5: Barriers and Opportunities in Optimizing Care

While all seven physicians successfully switched the majority of their patients off OxyContin, challenges were encountered. These included the lack of access to OxyNEO and the cost to those without drug plans, the abruptness of the discontinuation, and the short period that was allowed for physicians to switch patients to other pain medications. This pressured physicians to make conversions quickly, eliminating the possibility to wean patients gradually from OxyContin. While the rationale behind the discontinuation was considered reasonable, delays in notifying healthcare professionals about the discontinuation and in processing applications to receive OxyNEO were not. Anticipating a three-month period to process applications, physicians switched the majority of patients to other medications to avoid wait-times and withdrawal.

I thought it was done quite abruptly without any warning, I guess they had to do that because if there'd been warning, it's organized and there would have been OxyContin coming across the border and stuff…many of our patients only come every three months, so there was no way we could see all those patients and discuss with them, um, and the other thing was it was going to immediately convert to exceptional access so that, um, there was a little bit of a barrier, and then the 80 milligrams would not be available and also, um, so yeah time-wise, it wasn't enough time. I understand perhaps why they did it and I understand it was the company that set that timeline, not the provincial government or the pharmacies. (MD3)

In addition, physicians proposed the creation of a national database accessible by all healthcare providers to monitor patients' use of opioids and to prevent “doctor-shopping.” Some also stressed a need to provide additional training to all physicians to enhance opioid prescribing expertise.

## 4. Discussion

This study explored perceptions patients with chronic pain and their physicians had on OxyContin's discontinuation and its impact on care. Both patients and physicians agreed that gaps existed in the discontinuation process that could be improved on. Compared with physicians, areas of divergence for patients included the motive for discontinuation, the impact of discontinuation on chronic pain control, and the changes necessary to improve care.

Perceptions of the motivation for discontinuing OxyContin varied considerably among patients and physicians. Patients and physicians disagreed on the reason to discontinue OxyContin to address misuse and on whether phasing out the medication would fulfill this purpose. Despite doubting that restricting access to one problematic medication while leaving other opioids accessible would address the issue of addiction, physicians strongly agreed that the popularity of OxyContin among nonmedical users necessitated its discontinuation, asserting equally effective medications were available for legitimate users. In contrast, the majority of patients from this study believed that discontinuing OxyContin was wrong because it adversely affected their pain management and unfairly disadvantaged them. They had little faith that the strategy would work and held more negative beliefs regarding withdrawal and alternative treatment options. The introduction in 2010 of OxyNEO in the United States resulted in reduced abuse of OxyContin, but abuse of other long-acting opioid medications and heroin increased [28–30]. Such reports may have contributed to patient distrust in the effectiveness of discontinuation as a strategy to curb misuse. Furthermore, the introduction of generic versions of OxyContin sent a mixed, if not conflicted, message. Acknowledging the validity of these concerns and better explaining the evidence and rationale behind their own beliefs could help physicians to identify common ground with their patients, provide clarity, and, in turn, increase buy-in with making the transition.

Several other opportunities in patient-physician communication were identified. Patients were learning about OxyContin's discontinuation from the media rather than from their physicians. Physicians perceived that they had switched the great majority of their patients to other analgesics successfully, while patients felt more negatively impacted by the switch. Such differences of opinion were not openly communicated with each other. The short time to discontinuation, discontinuation that took place while some patients were between clinic visits, and the media announcement before physicians could take the time to inform their patients likely were all contributing factors. Patients were also unhappy about their lack of involvement in the decision-making process after discontinuation. While some patients prefer to participate in developing medical plans for their illnesses and others adopt a more passive attitude [27, 31], it has been shown that physicians who involve their patients in treatment decision-making report more satisfied patients than more authoritarian physicians [32].

Patients stated that physicians did not provide them with enough information and felt their concerns went unheard. For example, some patients found alternative medications following discontinuation were ineffective but felt they were unable to communicate such concerns. This is consistent with previous literature that showed clinicians can underestimate patient desire for information to better understand their health condition and their need to feel known and understood [33]. While incongruities between the clinicians' approach to illness and the patients' personal experiences of illness are well-documented [34–36], a patient-centered approach can help to minimize the impact and improve resulting care [37–39]. Using another example, some patients in this study expressed concerns over physical dependence and were interested in discontinuing opioid use. However, self-tapering resulted in withdrawal symptoms and negatively impacted their quality of life. Fear of addiction, tolerance, and adverse events are major concerns with opioid use [40]. An open, proactive discussion with patients regarding these concerns could help to initiate a safe, prescriber-guided opioid discontinuation plan.

Most patients and all physicians in this study felt that the healthcare system could improve but focused on different aspects. Patient dissatisfaction was most commonly related to not receiving adequate information about their medication therapy, particularly helping them to better understand the benefit-risk ratio at initiation. This was further complicated by conflicting messages from different healthcare professionals and the media. A helpful strategy could include dedicated time for standardized patient education for high-risk medications such as opioids at initial prescribing and/or dispensing. Integration of the pharmacist in this role could help to alleviate physician time constraints and reinforce key messages. On the other hand, most physicians admitted that managing opioid abuse against maintaining optimum pain control for chronic pain sufferers seems unattainable and required system-wide strategies. They supported proposals to develop a national database to better monitor the use of opioids [41, 42], to improve adherence to practice guidelines for opioid use, and to apply tools such as opioid contracts and urine screening tests [43–45]. Many of these are now a part of the action plans recently outlined by Health Canada and Ontario's Ministry of Health and Long-Term Care [46, 47]. In addition, Canadian evidence-based guidelines for opioid use, along with practice tools and decision-making aids, are in the process of being updated by 2017 [48–50]. These will be useful in guiding physicians and other clinicians towards safe and effective opioid prescribing and management. In addition, physicians advocated for earlier communication and a more reasonable timeline from policy makers. Both patients and physicians agreed that improved access to alternative agents to assist in the discontinuation process was essential.

While physicians generally expressed confidence in the ability of GPs to prescribe opioids, patients were more doubtful. A survey of Ontario primary care physicians showed that 86% felt confident with opioid prescribing but most voiced concerns over opioid use and 42% had patients who experienced opioid-related adverse events [49]. Other research showed pain specialists were more comfortable prescribing opioids than GPs, who typically had less training in opioid prescribing [51, 52]. That chronic pain, patient education, coordination of services, and interdisciplinary teamwork were often inadequately covered in medical curricula [53] can contribute to patients' negative experiences and/or lower physician comfort level. Reluctance of GPs to prescribe opioids might further compromise patient pain relief and QOL [23, 54]. Given that the majority of opioid prescribing occurs in primary care, education tools and resources designed to support primary care practice will be needed. Content on chronic pain management and opioid use should be better incorporated into core curriculum for physicians and other healthcare professionals. Enhancing patient education and patient-prescriber communication could also serve to increase patient confidence.

## 5. Strengths and Limitations

The diverse study sample of patients and physicians is a main strength of the study. Exploring experiences of physicians in different practice settings (i.e., GPs and pain specialists) as well as patients residing in rural and urban communities allowed us to collect data from varied perspectives. Secondly, obtaining perceptions from both patients and physicians illuminated certain points of agreement and controversy related to the same topic.

Inherent with voluntary participation, participant bias may have influenced the results. Participants with stronger opinions, whether positive or negative, may have been more likely to participate. The small sample also made the findings less generalizable to other population groups such as younger individuals with chronic pain and patients in other care settings such as long-term care. The lapse in time since discontinuation may have contributed to inaccuracies in recall. In addition, because of time and resource limitations, pharmacists were not included in the current study. Pharmacists play an important role in managing prescription opioid access and had first-hand experience in OxyContin's discontinuation.

## 6. Conclusion

This inquiry contributes to a better understanding of the first discontinuation and reformulation of an opioid indicated for chronic pain management in Canada. Patients and physicians disagreed in their level of support for OxyContin discontinuation and in their perception of the negative impact discontinuation had on pain control. They also identified numerous inadequacies within OxyContin's discontinuation and opioid prescribing processes that formed barriers to optimum care. Comprehending the patient and physician perspectives will help healthcare professionals and decision-makers better address these gaps in care.

## Figures and Tables

**Table 1:** General patient characteristics.

Patient	Age	Gender	Source of pain	OxyContin use	Care provider
A1	49	Female	Diabetic neuropathy	≈7 years	General practitioner
A2	51	Female	Chronic rotator cuff tears	≈8 years	General practitioner
A3	68	Female	Spinal stenosis	≈11 years	General practitioner
A4	52	Female	Occupational injury causing chronic back and leg pain	≈10 years	Pain specialist
B1	53	Female	Complex regional pain syndrome (lower back pain)	≈6 years	Pain specialist
B2	60	Female	Failed back syndrome	≈2 years	Pain specialist
B3	58	Female	Abdominal wall pain	≈7 years	Pain specialist
B4	52	Female	Fibromyalgia, osteoarthritis, restless leg syndrome	≈10 years	Pain specialist
B5	51	Male	Chronic rotator cuff tendinitis	≈5 years	Pain specialist
B6	46	Female	Severe painful sensory neuropathy	≈2 years	Pain specialist
C1	77	Female	Chronic low back pain	≈8 years	General practitioner
C2	78	Female	Osteoarthritis	≈4 years	General practitioner
C3	63	Female	Car accident causing chronic pain	≈8 years	General practitioner

**Table 2:** Findings from the Brief Pain Inventory (BPI).

Patient	Average pain score (0–10)	QOL domains with highest pain interference	Pain medications used since discontinuation of OxyContin
A1	2	Walking ability, appetite	OxyNEO, Gabapentin, Toradol, Tylenol
A2	4	Normal work, enjoyment of life	OxyNEO
A3	5	Normal work, enjoyment of life	Gabapentin, Tylenol
A4	8	Enjoyment of life	Percocet
B1	6	Sleep	OxyNEO, Percocet, Fentanyl Patch
B2	3	General activity, walking ability	OxyNEO
B3	6	General activity, sleep	OxyNEO
B4	7	Ability to concentrate, normal work, sleep	OxyNEO
B5	7	Sleep	OxyNEO
B6	8	Mood, normal work, sleep, ability to concentrate, enjoyment of life	OxyNEO, Lidocaine infusion
C1	5	Walking ability, normal work, sleep, enjoyment of life	OxyNEO, Fentanyl, Tylenol, Gabapentin
C2	1	Sleep	Hydromorphone
C3	6	Mood, normal work, sleep, ability to concentrate	OxyNEO

**Table 3:** Themes identified from patient interviews.

Themes	Subthemes							
(1) Disagreement with motive for discontinuation	Critical of authorities for discontinuing effective drug	Anticipate persistence of addiction problems	Feel irrationally deprived of pain medication	Address one issue by creating another	Blame prescribers for misuse and abuse	Question GPs ability to prescribe opioids	Discontinuation considered addicts not patients	Alternate solutions to addiction issues
(2) Discontinuation negatively impacted pain control	Optimal pain relief with OxyContin	Poorer pain management with substitutes	Endure trials of alternate medications	Experience withdrawal symptoms	Increased pain affecting cognition and function	Retrain self to manage new medications	Cost barrier to OxyNEO	
(3) Discontinuation insignificantly impacted pain control	Rate pain relief equivalent to before discontinuation	Continued receiving satisfactory pain control	Continued receiving inadequate pain control	Identification of differences in medications	Discontinuation has impact on other aspects			
(4) Choosing to get off OxyContin permanently	Fear lifelong dependence on OxyContin	Addiction to OxyContin driving decision	Tolerate withdrawal symptoms	Bear worsened pain	Reliance on distraction to ease pain			
(5) Learning to live with pain	Accepting life with pain	Recognize few alternatives	Wishing for a miracle drug	Hope to regain OxyContin				
(6) Barriers and opportunities in optimizing care	Learning about discontinuation from the media	Communication gap between patients and professionals	Feeling unheard by healthcare providers	Professionals not advising of addictive properties	Professionals not educating patients	Inadequate integration of general and pain clinics	Lack regulatory program to reassess pain	Not involving patients with decision-making

**Table 4:** Themes identified from physician interviews.

Theme	Subthemes			
(1) Support for the motive for discontinuation	Approve discontinuation	Support nonfunding and restricted access of OxyNEO	Addiction potential of OxyContin exceeds that of alternate drugs	Sufficient alternate drugs to OxyContin
(2) Discontinuation insignificantly impacted pain control	Patients attain similar pain relief with alternate medications	Transition to other drugs carried out efficiently	Sufficient alternate drugs to choose from	Unsatisfactory outcome ascribed to medication abuse
(3) Sufficient resources for opioid prescribing	Appropriate prescribing of opioids results in safe and effective use	Lack of adherence to existing guidelines is the real issue	Narcotic database will enhance monitoring strategies	
(4) Disapproval of generics	Existing pain medications are adequate	Introduction of generics defeats discontinuation	Physicians do not prescribe generics	Generics drug of choice for addicts
(5) Barriers and opportunities in optimizing care	Switching to a different medication was successfully accomplished	Exceptional Access Program made it extremely difficult to access OxyNEO	Abruptness of the change with short notice period to change drugs	Delay in notifying professionals and in processing applications for patients to start receiving OxyNEO

## Additional Points

*Summary.* OxyContin was one of the most commonly prescribed medications for chronic pain in Canada. It was delisted from the Ontario Drug Benefit Formulary in 2012 because of concerns of addiction and misuse. This qualitative study explores the impact of discontinuing OxyContin on chronic pain patients formerly prescribed it, as elucidated by interviews with patients and their treating physicians. Patient views differed markedly from those of their physicians on the motive to discontinue OxyContin, perceived impact of discontinuation on pain control, and deficiencies within the healthcare system. Patient and physician views were congruent on the need to optimize current pain management practices.
